# Myostatin Inhibits Vascular Smooth Muscle Cell Proliferation and Local 14q32 microRNA Expression, But Not Systemic Inflammation or Restenosis

**DOI:** 10.3390/ijms21103508

**Published:** 2020-05-15

**Authors:** Eveline A.C. Goossens, Margreet R. de Vries, J. Wouter Jukema, Paul H.A. Quax, A. Yaël Nossent

**Affiliations:** 1 Department of Surgery, Leiden University Medical Center, 2300 RC Leiden, The Netherlands; e.a.c.goossens@lumc.nl (E.A.C.G.); m.r.de_vries@lumc.nl (M.R.d.V.);; 2 Einthoven Laboratory for Experimental Medicine, Leiden University Medical Center, 2300 RC Leiden, The Netherlands; 3 Department of Cardiology, Leiden University Medical Center, 2300 RC Leiden, The Netherlands; j.w.jukema@lumc.nl; 4 Department of Internal Medicine II, Medical University of Vienna, 1090 Vienna, Austria; 5 Department of Laboratory Medicine, Medical University of Vienna, 1090 Vienna, Austria

**Keywords:** myostatin, 14q32 locus, microRNAs, restenosis, postinterventional vascular remodelling

## Abstract

Myostatin is a negative regulator of muscle cell growth and proliferation. Furthermore, myostatin directly affects the expression of 14q32 microRNAs by binding the 14q32 locus. Direct inhibition of 14q32 microRNA miR-495-3p decreased postinterventional restenosis via inhibition of both vascular smooth muscle cell (VSMC) proliferation and local inflammation. Here, we aimed to investigate the effects of myostatin in a mouse model for postinterventional restenosis. In VSMCs in vitro, myostatin led to the dose-specific downregulation of 14q32 microRNAs miR-433-3p, miR-494-3p, and miR-495-3p. VSMC proliferation was inhibited, where cell migration and viability remained unaffected. In a murine postinterventional restenosis model, myostatin infusion did not decrease restenosis, neointimal area, or lumen stenosis. Myostatin inhibited expression of both proliferation marker PCNA and of 14q32 microRNAs miR-433-3p, miR-494-3p, and miR-495-3p dose-specifically in cuffed femoral arteries. However, 14q32 microRNA expression remained unaffected in macrophages and macrophage activation as well as macrophage influx into lesions were not decreased. In conclusion, myostatin did not affect postinterventional restenosis. Although myostatin inhibits 14q32 microRNA expression and proliferation in VSMCs, myostatin had no effect on macrophage activation and infiltration. Our findings underline that restenosis is driven by both VSMC proliferation and local inflammation. Targeting only one of these components is insufficient to prevent restenosis.

## 1. Introduction

Atherosclerotic occlusions of the coronary artery and the femoral artery, causing coronary artery disease (CAD) and peripheral artery disease (PAD) respectively, are often treated by balloon angioplasty with or without stenting [1,2,3,4]. However, in both CAD and PAD, restenosis often occurs due to a variety of factors [5]. Even when pro-atherogenic risk factors, such as hypercholesterolemia and diabetes mellitus, are strictly controlled, restenosis can still develop. Upon physical manipulation of the occluded vessel during the angioplasty, the vascular wall becomes activated. On a cellular level, two vascular responses are crucial [5,6]. On the one hand, vascular smooth muscle cells (VSMCs) that reside in the tunica media of the arterial wall change their phenotype from contractile to synthetic, meaning that they start to proliferate and migrate into the tunica intima, forming a neointimal layer. On the other hand, inflammatory cells adhere to and infiltrate the affected vessel wall. Of these inflammatory cells, monocytes/macrophages are among the first to arrive and drive the inflammatory response. Inflammation allows for extracellular matrix remodelling, further facilitating the intimal hyperplasia. Although initiated locally, restenosis is driven by both local and systemic inflammation. 

Patients with an atherosclerotic occlusion that are treated with balloon angioplasty, often receive drug-eluting stents (DES) that secrete drugs locally to inhibit restenosis [7,8]. Many studies have been performed to find the most effective compound to reduce restenosis, focusing on anti-proliferative drugs. However, some compounds also target the inflammatory side of restenosis [9]. Sirolimus and paclitaxel are among the most frequently used compounds in DES. Sirolimus (rapamycin) has antiproliferative properties and is an immunosuppressing agent, inhibiting the local inflammatory reaction in restenosis [10]. Paclitaxel is antimitotic and therefore strongly antiproliferative in VSMCs [11], but does not act on inflammation and the therapeutic window is narrow [12]. Nowadays, DES that are mostly used, are sirolimus-like stents. Short term clinical outcomes between the two stents are similar, but long-term efficacy and safety favor sirolimus stents [12,13]. Despite the existing stents however, restenosis remains a common issue in the fields of cardiology and vascular surgery and more efficient therapeutic strategies are still needed.

Myostatin, also known as Growth Differentiation Factor-8 (GDF-8), is a member of the TGF-beta superfamily that negatively regulates skeletal muscle mass by inhibiting muscle hypertrophy and hyperplasia; myostatin null mice show a dramatically increased skeletal muscle mass [14]. Accordingly, administration of myostatin causes muscle atrophy [15]. Myostatin is not only expressed in skeletal muscle cells, but also in cardiomyocytes and VSMCs [16,17]. Although myostatin was shown to affect muscle cell function via extracellular binding to the activin type 2 receptor [18], intracellular effects, in which myostatin directly affects gene transcription, were also observed [19]. One of the genomic regions that is affected by myostatin is called the callipyge locus, also known as the 14q32 locus in humans. Callipyge originates from ancient Greek and means beautiful buttocks. Both absence of myostatin [19] and mutations in the 14q32 locus [20] can lead to a ‘callipyge phenotype’, which is defined by excessive muscle growth in cattle, sheep and mice. The callipyge locus contains three protein coding genes, but also a large conserved microRNA cluster, the 14q32 microRNA cluster (DIO3-DLK1 cluster, 12F1 in mice) that is known to play a role in many different vascular remodelling processes [21,22,23,24,25]. Upon knockout of myostatin in mice, microRNAs of the 12F1 locus were upregulated [19]. 

MicroRNAs are short non-coding RNA molecules that regulate gene expression by binding to the 3’-UTR of their target messenger RNA (mRNA), thereby inhibiting translation. As one microRNA has the ability to bind to multiple target genes, a single microRNA can affect entire (patho)physiological processes. This makes them promising targets in vascular remodelling, which is always a multifactorial process. In 2017, our group found that direct inhibition of 14q32 microRNA miR-495-3p reduces intimal hyperplasia, macrophage influx and overall lesion formation in experimental restenosis [21]. As myostatin has the potential to inhibit both VSMC proliferation and the expression of miR-495-3p, as well as other 14q32 microRNAs, simultaneously, we hypothesized that administration of myostatin will reduce postinterventional restenosis.

In this study, we aimed to investigate the role of myostatin in postinterventional restenosis, making use of an established murine restenosis model [12,21,26,27,28,29]. In this model, a non-constrictive cuff is placed around both femoral arteries. Manipulation of the artery, as well as a foreign body response to the cuff, triggers both intimal hyperplasia and an inflammatory response [30]. We focused on the effects of myostatin infusion on VSMC proliferation, neointima formation, on macrophage infiltration into the lesions and of course on 14q32 microRNA expression. We looked specifically at miR-433-3p, miR-494-3p, and miR-495-3p, as the genes encoding these microRNAs are located separately along the length of 14q32 locus, as it was previously shown that myostatin affects expression of the complete locus, rather than single microRNAs [19].

## 2. Results

### 2.1. In Vitro Uptake of Myostatin in VSMC

**Figure 1:**
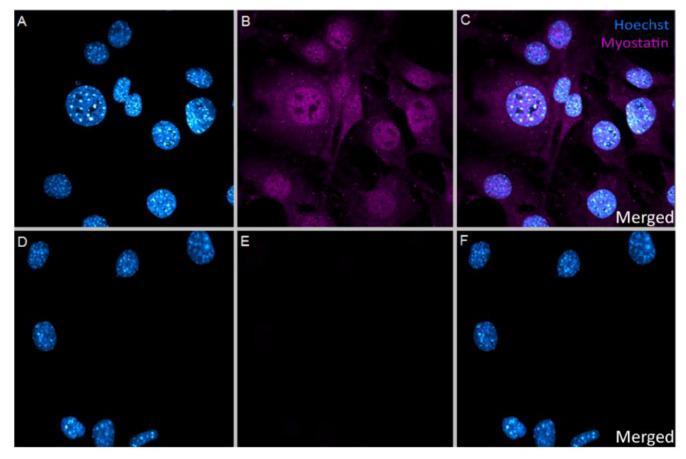
(**A**–**F**): Immunocytochemical staining of VSMCs. (**A**–**C**)—VSMCs treated with 10 nM myostatin showed uptake of myostatin in the cell, concentrating mostly in the nucleus. (**A**)—Hoechst only, (**B**)—Myostatin only, (**C**)—Merged image. (**D**–**F**)—VSMCs not treated with myostatin showed no endogenous myostatin presence in the cells at all. (**D**)—Hoechst only, (**E**)—Myostatin only, (**F**)—Merged image. Picture taken with 63× magnification.

Since myostatin can bind either to the cell surface receptor activin type II or can enter the nucleus, we first identified in vitro whether myostatin is taken up by VSMCs and, more specifically, is present in the nucleus. As shown in Figure 1A–C, myostatin was taken up into the VSMCs, concentrating mostly inside the nucleus. The endogenous expression of myostatin in the resting VSMC was negligible as in the negative control, where no myostatin was added, myostatin staining was absent (Figure 1D–F).

### 2.2. Effect of Myostatin on 14q32 microRNAs in VSMCs

**Figure 2:**
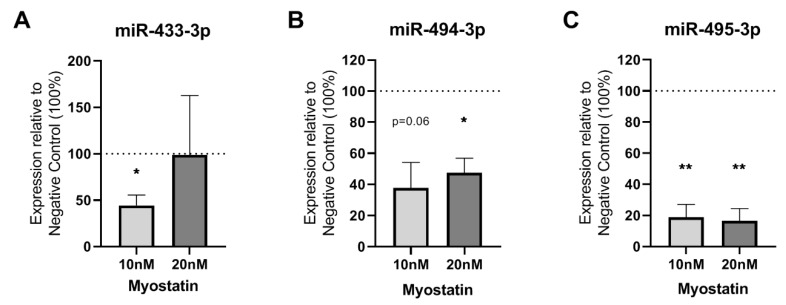
(**A**–**C**): microRNAs in myostatin treated VSMCs relative to untreated control (100%). (**A**)—miR-433-3p expression in VSMCs treated with 10 nM and 20 nM myostatin only showed a downregulation at 10 nM treatment (*p* = 0.04). (**B**)—miR-494-3p expression in VSMCs treated with 10 and 20 nM myostatin was downregulated at 20 nM treatment (*p* = 0.03) and showed a trend towards downregulation at 10 nM myostatin (*p* = 0.06). (**C**)—miR-495-3p expression in VSMCs treated with 10 and 20 nM myostatin was decreased with more than 80% by both treatment concentrations (*p* = 0.0098 and *p* = 0.0088, respectively). Mean expression is shown and error bar represents SEM (*n* = 3). One-sample *t*-test performed with 100% of control expression as hypothetical value. * *p* < 0.05, ** *p* < 0.01.

We analyzed the effects of myostatin on expression levels of the specific 14q32 microRNAs miR-433-3p, miR-494-3p and miR-495-3p. The addition of 10 or 20 nM recombinant myostatin to VSMCs affected microRNA expression levels. For miR-433-3p, 10 nM of myostatin downregulated microRNA expression (*p* = 0.04), but 20 nM did not affect miR-433-3p expression (Figure 2A). MiR-494-3p showed a downregulation after treatment with both 10 nM and at 20 nM myostatin (Figure 2B). MiR-495-3p was also downregulated by more than 80% after both 10 and 20 nM myostatin treatment (*p* = 0.01 and *p* = 0.01, respectively) (Figure 2C).

### 2.3. Functional Effects of MSTN on VSMCs

**Figure 3:**
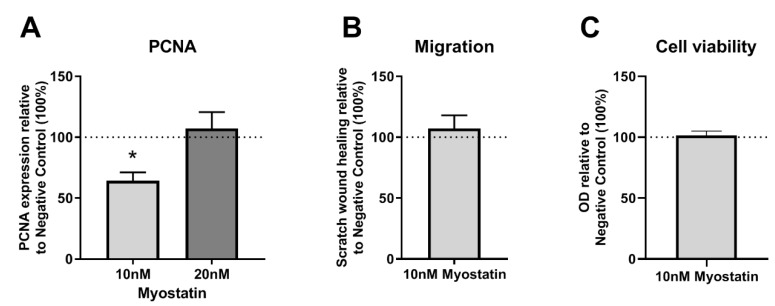
(**A**–**C**): PCNA expression, cell migration and cell viability in myostatin treated VSMCs relative to non-myostatin treated control (100%). (**A**)—PCNA mRNA expression in myostatin treated VSMCs was downregulated at 10 nM myostatin, but not at 20 nM myostatin, *n* = 3. (**B**)—Scratch wound healing in myostatin treated VSMCs was not changed, *n* = 3. (**C**)—Cell viability in myostatin treated VSMCs remained unchanged, *n* = 4. Mean expression is shown and error bars represent SEMs. One-sample t-test performed with 100% of Negative Control expression as control group.

Proliferating Cell Nuclear Antigen (PCNA) mRNA expression, a measure of cell proliferation, was downregulated by approximately 40% when VSMCs were treated with 10 nM myostatin compared to untreated cells (*p* = 0.04) (Figure 3A). However, the addition of 20 nM myostatin did not result in the downregulation of PCNA expression in cultured VSMCs. Migration and cell viability remained unaffected by 10 nM myostatin, compared to the negative control (Figure 3B,C).

### 2.4. Restenosis in Myostatin-Treated Mice

**Figure 4:**
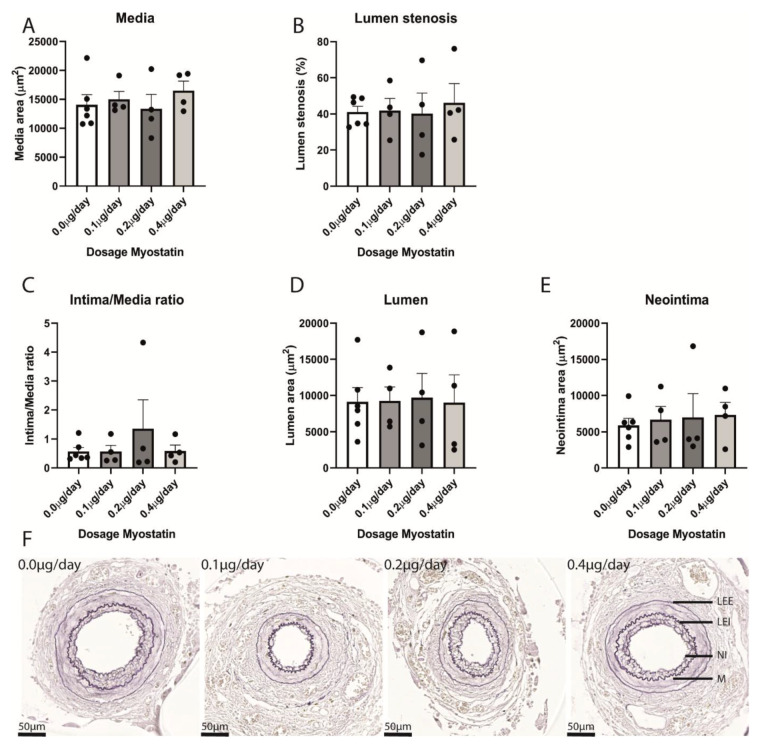
(**A**–**F**): Quantification of restenosis in cuffed femoral arteries in different treatment groups. (**A**)—Media area was similar in different treatment groups. (**B**)—Lumen stenosis did not differ between treatment groups. (**C**)—Intima/Media ratio was similar in different treatment groups. (**D**)—Lumen area in different treatment groups was equal. (**E**)—Neointima area in different treatment groups did not differ. (**B**–**E**): Quantification of not occluded arteries. Mean expression is shown and error bar represents SEM. *n* = 7 in control group, *n* = 4 in 0.1 µg/day, *n* = 4 in 0.2 µg/day, *n* = 4 in 0.4 µg/day. Student’s t-test, performed between myostatin treated groups and control group with α = 0.05, did not show any significant differences. (**F**)—representative examples of cuffed femoral arteries in different treatment groups. LEE = Lamina Elastica Externa, LEI = Lamina Elastica Interna, NI = Neointima, M = Media.

As there appeared to be a rest restricted therapeutic window in myostatin efficacy in vitro, we used multiple myostatin dosages in vivo. The highest dose of 0.4 µg/day was based on previously published work by others [31]. With the dose-specific effect in vitro, we choose to also include two groups of mice treated with either 0.1 or 0.2 µg/day. Mice subjected to femoral artery cuff placement thus received 0.0 (control group), 0.1, 0.2 or 0.4 µg/day of myostatin via continuous infusion from an osmotic pump. We analyzed intimal hyperplasia and restenosis in C57BL/6 mice that were sacrificed three weeks after femoral artery cuff placement. Arteries that showed 100% stenosis were excluded from the analyses (Supplementary Figure S1). In the remaining arteries, the medial layer area was similar in all groups (Figure 4A). Lumen stenosis and intima-media ratio did not differ either (Figure 4B,C). Finally, lumen area and neointima area were also similar in all groups (Figure 4D,E). Figure 4F shows representative femoral arteries with restenosis formation for each group.

### 2.5. Myostatin in Cuffed Femoral Arteries

**Figure 5:**
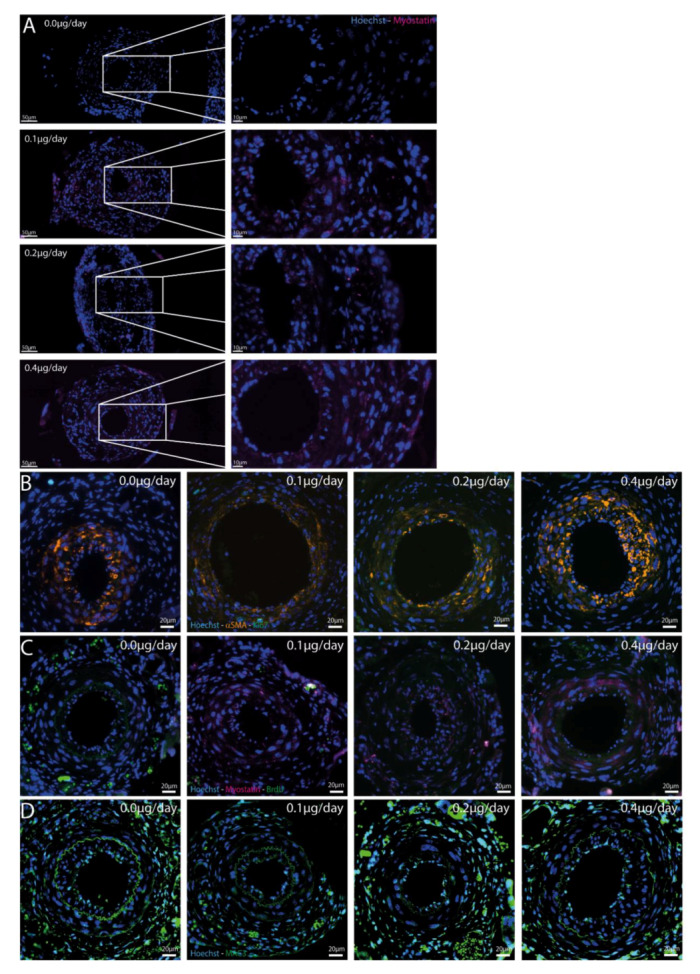
(**A**–**D**): Immunofluorescent stainings in different treatment groups. (**A**)—Myostatin-Hoechst immunofluorescent double staining. Representative mouse of every treatment group is shown and zoom-in of femoral artery shows presence of myostatin in all treated groups, but not in the control group. (**B**)—Ki-67-αSMA-Hoechst immunofluorescent triple staining in cuffed femoral arteries of different treatment groups shows few proliferating cells, especially not in the αSMA area. (**C**)—BrdU-Myostatin-Hoechst immunofluorescent triple staining in cuffed femoral arteries of different treatment groups shows again little proliferating cells and those cells are not double stained with myostatin. (**D**)—Hoechst-MAC3 immunofluorescent double staining of cuffed femoral arteries showed macrophages in all groups in both the intimal and medial layers, but no differences were found.

No endogenous myostatin expression was measured in the femoral arteries in any of the groups (data not shown). However, as shown in Figure 5A, the infused myostatin was effectively taken up by the cuffed arteries at all three dosages, where no myostatin was present in the control group.

### 2.6. Effect of Myostatin Treatment on Proliferation

**Figure 6:**
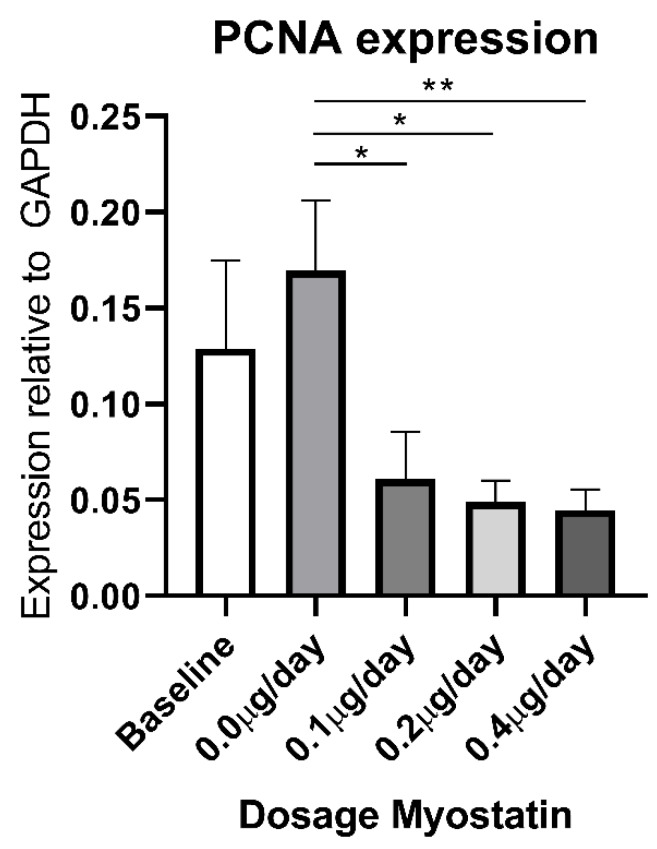
PCNA mRNA expression in femoral arteries of myostatin treated mice. PCNA mRNA expression is significantly lower in myostatin treated groups at all dosages compared to the control group. Mean expression is shown and error bar represents SEM (*n* = 8 for 0.0, 0.1 and 0.4 µg/day myostatin group, *n* = 6 for baseline and 0.2 µg/day group). Student’s t-test performed between treated groups and control group with α = 0.05. * *p* < 0.05, ** *p* < 0.01.

We then stained the cuffed femoral arteries for Ki-67, however hardly any positive cells were observed within the medial and intimal regions in any of the arteries (average of one cell/section) (Figure 5B). These Ki-67 positive cells stained negative for αSMA, indicating that VSMCs are not the cells that are still proliferating at this late time point (three weeks after cuff placement). Moreover, we performed a BrdU staining and again observed no proliferating cells in the neointimal layer or medial layer of the femoral arteries (Figure 5C). PCNA mRNA expression, however, was decreased in myostatin-treated mice compared to controls (Figure 6).

### 2.7. Effect of Myostatin on Macrophages

**Figure 7:**
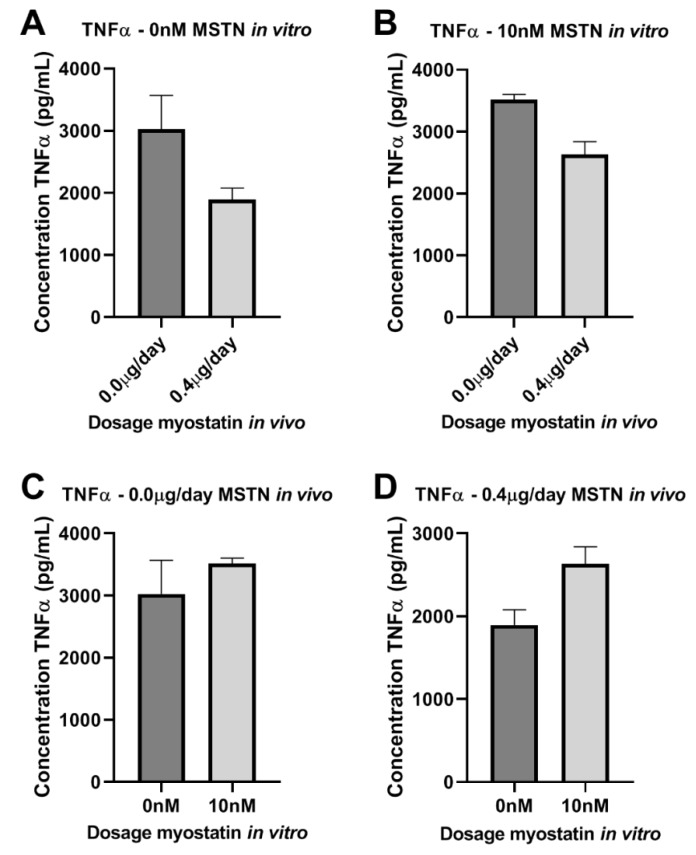
(**A**–**D**): Effect of myostatin on macrophages. (**A**–**D**)—TNFα in supernatant of bone marrow macrophages stimulated with myostatin. (**A**,**B**)—in vivo treated mice with 0.0 vs. 0.4µg myostatin/day and not treated in vitro with myostatin (**A**) or in vitro treated with 10 nM myostatin (**B**) did not show significant differences. (**C**,**D**)—in vivo not treated (**C**) or treated (**D**) and in vitro not treated or treated with myostatin did not show significant differences. Mean expression is shown as average of duplicate measurements of pooled cells and error bar represents SEM. Student’s t-test did not show significant differences.

We then stained for MAC3 and observed that macrophages were present in both the neointimal and the medial layers of the cuffed femoral arteries in all mice (Figure 5D). However, the percentages of macrophages in the intimal layer, the medial layer and in both layers together, did not differ between groups (Supplementary Figure S2A–C). To further analyze the effect of myostatin on macrophage activation, isolated bone marrow monocytes of four control mice and four 0.4 µg myostatin/day-treated mice were pooled per group and subjected to either 10 nM additional myostatin in culture medium or culture medium only during maturation into macrophages in vitro. After maturation, macrophages were stimulated with 10 ng/mL LPS for 48 h and TNFα level was measured in the medium. TNFα secretion appeared lower in macrophages from myostatin-treated mice than from untreated mice, independent of the additional in vitro treatment with myostatin during the culturing period (Figure 7A,B). However, when we looked within the myostatin-treated or untreated mice, TNFα secretion appeared higher when myostatin was added during in vitro maturation of monocytes into macrophages (Figure 7C,D). It should be noted that neither effect was strong enough to detect any statistically significant differences.

### 2.8. Effect of Myostatin Treatment on 14q32 microRNA Expression In Vivo

**Figure 8:**
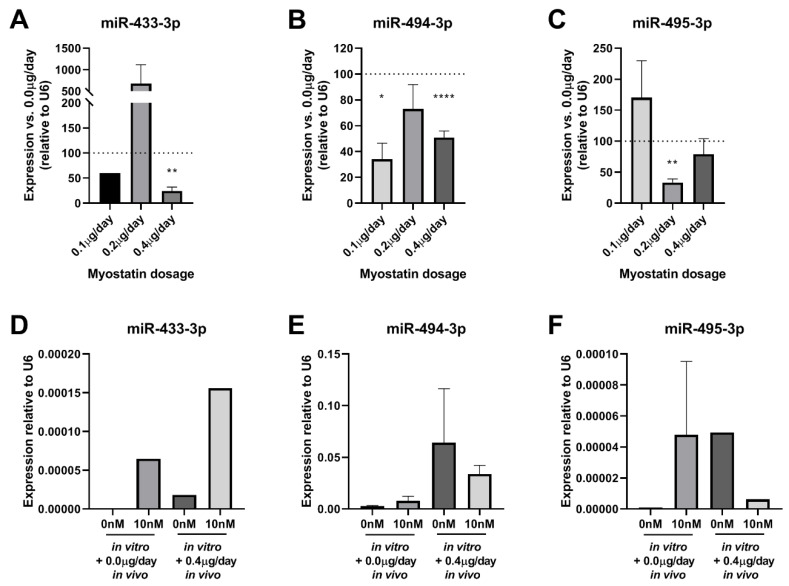
(**A**–**F)**: microRNA expression in femoral arteries and macrophages. (**A**)—miR-433-3p expression in femoral arteries of 0.1, 0.2, 0.4 µg/day of myostatin relative to control mice (100%) showed significant downregulation of miR-433-3p at 0.4 µg/day (*p* = 0.003). (**B**)—miR-494-3p expression in femoral arteries of 0.1, 0.2, 0.4 µg/day of myostatin relative to control mice (100%) showed significant downregulation of miR-494-3p at 0.1 (*p* = 0.001) and 0.4 µg/day (*p* < 0.0001). (**C**)—miR-495-3p expression in femoral arteries of 0.1, 0.2, 0.4 µg/day of myostatin relative to control mice (100%) showed significant downregulation of miR-495-3p at 0.2µg/day (*p* = 0.0003). For (**A**–C) Mean expression is shown and error bars represent SEM (*n* = 8 for 0.0, 0.1 and 0.4 treatment groups, *n* = 6 for 0.2 treatment group). One-sample t-test performed with 100% expression of control group. * *p* < 0.05, ** *p* < 0.01, **** *p* < 0.0001. (**D**)—miR-433-3p expression did not show any differences between groups. (**E**)—miR-494-3p expression did not show any differences between groups. (**F**)—miR-495-3p expression did not show any differences. For (**D**–**E**) bone marrow monocytes matured into macrophages from in vivo treated mice with 0.0 vs. 0.4 µg myostatin/day and in vitro treated without myostatin or with 10 nM myostatin. Maturated cells were stimulated with LPS to trigger an inflammatory reaction. Mean expression is shown as average of duplicate measurements of pooled macrophages and error bar represents SEM. Wilcoxon rank sum test did not show any differences.

We assessed the effects of myostatin treatment on microRNA expression in vivo compared to the control group. As shown in Figure 8A–C, in the cuffed femoral arteries levels of the 14q32 microRNAs miR-433-3p, miR-494-3p, and miR-495-3p were downregulated in response to myostatin, but again, only at specific dosages. For miR-433-3p, 0.4 µg myostatin/day resulted in downregulation (*p* = 0.003, Figure 8A), but at other dosages, no effect was observed. MiR-494-3p, however, showed decreased expression in both 0.1 µg myostatin/day (*p* = 0.001) and 0.4 µg myostatin/day groups (*p* < 0.0001, Figure 8B). MiR-495-3p expression level was downregulated by 50% in the 0.2 µg myostatin/day treatment group (*p* = 0.0003, Figure 8C). No miR-495 downregulation was observed in the 0.1 µg/day and for 0.4 µg/day myostatin groups (Figure 8C).

MicroRNA expression levels in bone marrow-derived macrophages were not downregulated in any of the treatment groups, i.e., long-term in vivo treatment with myostatin and control or in vitro treatment and control for any of the measured 14q32 microRNAs (Figure 8D–F). 

## 3. Discussion

In this study, myostatin was investigated as regulator of VSMC proliferation and 14q32 microRNA expression with the ultimate goal to inhibit postinterventional restenosis. As anticipated, VSMC proliferation was inhibited and 14q32 microRNAs were downregulated in response to myostatin treatment, however, there were no effects on restenosis.

Myostatin is known to act mainly on skeletal muscle cells to inhibit their proliferation and growth [14,15]. However, the facts that myostatin also functions in VSMCs [17] and that, in restenosis, exactly these VSMCs proliferate and migrate to form a neointimal layer [32], suggest that myostatin treatment could be a promising therapeutic compound for this important clinical problem. PCNA expression was downregulated by myostatin treatment both in vitro and in vivo, indicating that cell proliferation was indeed reduced by myostatin, where cell viability and migration remained unaffected. In contrast to the reduction in in vivo PCNA levels however, BrdU and Ki-67 were hardly detectable anymore in either the neointimal layer or the medial layer in any of the groups, indicating that, at the time of sacrifice, no active cell proliferation was ongoing in the lesions anymore. We can only speculate on the reasons for this discrepancy, however, most likely, the process of neointima formation is already complete after three weeks in this particular model. To better understand this, in future studies, one may choose to sacrifice the mice at earlier time points. Or one may trace the origin of the cells in the neointima, to determine cell migration rather than proliferation may also play a role. However, as myostatin clearly did not affect the final outcome, we have not included such experiments here.

It was previously shown that myostatin administration via injection of a Chinese hamster ovarian (CHO) cell line that overexpresses murine myostatin in athymic nude mice, compared to CHO-control cell injection, leads to muscle atrophy [15], which would be detrimental of course for any potential therapeutic compound. However, at the dosages used in this study, we did not observe any adverse effects of myostatin infusion. The arterial medial layers appeared normal in all groups, suggesting that VSMC-specific cytotoxic effects did not occur, which corresponds with our in vitro observation that myostatin did not affect cell viability. Furthermore, the elastic laminas, that in case of toxic side effects may show typical breaks [12], were intact in all patent vessels, confirming the absence of toxic effects.

As anticipated, myostatin treatment resulted in downregulation of 14q32 microRNA miR-433-3p, miR-494-3p and miR-495-3p expression, both in vitro in VSMCs and in vivo in the arterial wall of which VSMCs are the major cellular component. This fits with previous findings of myostatin’s actions on the callipyge locus [19]. Both in vitro and in vivo we found that there is a defined therapeutic window however, with a dose-specific effect of myostatin on 14q32 microRNA downregulation in VSMCs. In macrophages, microRNA expression levels remained unaffected by myostatin treatment. As described previously, microRNAs show highly cell-type specific expression patterns [33] and have cell-type specific effects [34], which we confirmed in the present study.

The effect of myostatin on macrophages was further assessed in the cuffed femoral arteries. We did not observe differences in influx of macrophages in any of the treatment groups. This implies that myostatin does not affect migration and activation of macrophages in our restenosis model. Moreover, the assessment of bone marrow-derived macrophages from myostatin-treated and control mice did not show any clear trend towards increased or decreased macrophage activation. Together with the lack of effect of myostatin on microRNA expression in these macrophages, we conclude that myostatin does not lead to sufficient changes in macrophage activation to contribute to a reduction in local inflammation in the artery and thus to a reduction in restenosis.

It has been demonstrated that 14q32 microRNAs act in multiple forms of vascular remodelling. Previous experimental studies showed that inhibition of 14q32 microRNAs decreases atherosclerosis and stimulates angiogenesis, but also decreases restenosis [21,23,25]. Thus, lower expression of 14q32 microRNAs stimulates beneficial remodelling and reduces maladaptive processes. In cardiovascular disease, this implies that downregulation of 14q32 microRNAs results in advantageous vascular remodelling. We anticipated that decreased levels of 14q32 microRNAs, as a result of myostatin infusion, would therefore lead to a reduction in intimal hyperplasia and restenosis in our model. This was not the case, however. We have previously shown that systemic inhibition of a single 14q32 microRNA, miR-495-3p, which was also downregulated in the femoral artery wall in the current study, resulted in a significant reduction in restenosis [21]. However, after systemic miR-495-3p inhibition, we also observed a reduction in the number of macrophages that infiltrated the lesions. Myostatin reduces miR-495-3p, as well as other 14q32 microRNAs, but in VSMCs only. Clearly, 14q32 microRNAs act on multiple cell types and tissues and inhibition in VSMCs alone is not enough to reduce maladaptive changes in the arterial wall. 

Taken together, our findings demonstrate that systemically infused myostatin acts locally in the arterial wall to downregulate intracellular 14q32 microRNA expression and decreases VSMC proliferation, but myostatin does not decrease 14q32 microRNA expression levels in macrophages, nor does it affect their activation or infiltration in the arterial wall. As we could not decrease postinterventional restenosis, our myostatin study emphasizes the need to target both VSMC proliferation and inflammation in restenosis. Of the drugs available in the clinic nowadays, sirolimus is the only one that affects both VSMC proliferation and the inflammatory side of restenosis [10]. Other drug-eluting stents, like paclitaxel-eluting stents, only inhibit proliferation and are therefore less favorable [13].

## 4. Materials and Methods

### 4.1. Animals and Femoral ArteRy Cuff Mouse Model

This study was performed in compliance with Dutch government guidelines and the Directive 2010/63/EU of the European Parliament. All animal experiments were approved by the animal welfare committee of the Leiden University Medical Center (study number 1160020172409_18.393). Male C57BL/6 mice (8–10 weeks old) with unrestricted access to food and water were used. Mice were randomized into groups based on age and weight. As described previously [21], mice underwent bilateral cuff surgery under adequate anesthesia and peri-operative analgesia, i.e., 5 mg/kg midazolam (Roche Diagnostics, Almere, the Netherlands), 0.05 mg/kg fentanyl (Janssen Pharmaceuticals, Leiden, the Netherlands), and 0.5 mg/kg dexmedetomidine (Orion, Mechelen, Belgium). After the incision, the iliac fat pad was located and the femoral artery was separated from the femoral vein and the femoral nerve and the non-constrictive polyethylene cuff was put into place and fixed with 2 6/0 sutures. The skin was closed with a continuous suture. Three groups of mice received Alzet Osmotic Pumps with myostatin (R&D systems, cat#788-G8/CF, Minneapolis, MI, USA) in administration concentrations of 0.4, 0.2 and 0.1 µg/day and the control group received mini pumps with vehicle. Dosage of 0.4 µg/day was described in literature to not have unwanted side effects as affecting body weight [31]. With the dose specific effect in vitro, we also choose to use 0.1 and 0.2 µg/day treatments. The pumps were placed subcutaneously in the neck and the skin was closed with two wound clips. Depending on the wound closure rate, at day 7–10, mice underwent Isoflurane anesthesia to remove the wound clips. In 0.2 µg/day group, two mice died postoperatively. In 0.1 µg/day group one mouse was taken out of the study as we observed during histological analysis that the femoral artery was not completely located in the cuff. At day 18, 19, 20 after surgery 200 µL BrdU (5 mg/mL) was injected i.p. for labeling of proliferating cells. On the day of sacrifice (21 days after surgery), the animals underwent terminal anesthesia, blood was drawn via heart puncture, after which a perfusion with PBS and formaldehyde was applied. Next, the cuffed arteries and hindlimb bones were collected for further processing.

### 4.2. Cell Culture

Immortalized VSMCs [35] were cultured at 37 °C in a humidified 5% CO_2_ enviro nMent. Culture medium (DMEM GlutaMAX™ (GIBCO, Thermo Fisher Scientific, Waltham, MA, USA)), 10% heat inactivated fetal calf serum (PAA), 1% penicillin (10,000 U/mL)/streptomycin (10,000 U/mL)) was refreshed every 2–3 days. Cells were passed at 90% confluency using trypsin (Sigma-Aldrich, Zwijndrecht, The Netherlands). Stock solutions of isolated VSMCs up to passage four were stored at –180 °C in 50% DMEM GlutaMAX™ containing 10% FCSi and 1% Penicillin/Streptomycin, 40% FCSi (PAA) and 10% DMSO (Sigma).

### 4.3. In Vitro Addition of Myostatin to VSMCs

VSMCs were seeded in 12-well plates at 60,000 cells per well in culture medium. After 24 h, cells were washed with PBS and each well was incubated with starve medium (DMEM GlutaMAX™ (Invitrogen, GIBCO) with 2% fetal bovine serum and 1% penicillin (10,000 U/mL)/streptomycin (10,000 U/mL) with or without recombinant myostatin (R&D systems, cat#788-G8) for 48 h in different concentrations, 10 and 20 nM. For further microRNA analyses medium was aspired and cells were washed with PBS before adding 0.5 mL TRIzol/well for RNA isolation. Each condition was performed in triplicate and each experiment was performed at least three times.

### 4.4. Scratch-Wound Healing Assay

After 48 h of incubation with 10 nM recombinant myostatin or without recombinant myostatin medium was aspired and a scratch-wound was made across the diameter of each well using a p200 pipet tip. Next, cells were washed with PBS and fresh medium (DMEM GlutaMAX™ (Invitrogen, GIBCO)), 2% FBS, 1% penicillin (10,000 U/mL)/streptomycin (10,000 U/mL)) was added. In order to monitor scratch-wound closure, live phasecontrast microscropy (Axiovert 40C, Carl Zeiss Oberkochen, Germany) was used for taking pictures immediately after (0 h) and 21 h after introducing the scratch-wound. Pictures were taken at two different locations in each well and averaged for analysis. Scratch size was calculated at 0 and 21 h using the wound healing tool macro for ImageJ. Each single scratch assay condition was performed in triplicate and the scratch-wound healing assay was performed three times.

### 4.5. MTT Viability Assay

VSMCs were seeded in 96-wells plate with 4000 cells/well. After 24 h, medium was aspired and cells were washed with PBS. Starve medium with 10 and 20 nM recombinant myostatin or without myostatin was added and cells were incubated for 48 h. Negative control was culture medium with 10% DMSO as toxic agent and positive control was culture medium. 10 µL of MTT (5 mg/mL) was added and after 4 h of incubation medium was removed carefully and replaced by isopropanol 0.1 N HCl. Plate was incubated on shaker platform for 90 m at 250 rpm and absorbance was read at 540 nM using Cytation5 (BioTek, Winooski, VT, USA). Each single scratch assay condition was performed in quadruplicate and the viability assay was performed four times.

### 4.6. RNA Isolation

Murine tissues were homogenized in TRIzol with electric pestle before starting RNA isolation. RNA isolation of both cultured cells and murine tissue was performed by standard TRIzol-chloroform extraction, according to the manufacturer’s instructions (Thermo Fisher Scientific, Waltham, MA, USA). RNA concentrations were measured using Nanodrop^TM^ 1000 Spectrophotometer (Thermo Fisher Scientific). 

### 4.7. MicroRNA Quantification

For microRNA quantification of miR-433-3p, miR-494-3p, and miR-495-3p, in all samples, RNA was reversed transcribed using the Taqman^TM^ MicroRNA Reverse Transcription Kit (Thermo Fisher Scientific) and subsequently quantified using microRNA-specific Taqman^TM^ qPCR kits (Thermo Fisher Scientific) on the VIIa7 (Thermo Fisher Scientific). MicroRNA expression was normalized against U6 small nuclear RNA. 

### 4.8. mRNA Quantification

For quantification of mRNA, RNA was reverse transcribed using ‘high-capacity RNA to cDNA kit’ (Thermo Fisher Scientific) and quantified by qPCR using SybrGreen reagents (Qiagen) on the VIIa7. Primer sequences are provided in Supplementary Table S1.

### 4.9. Immunocytochemistry

VSMCs were plated on 0.2% gelatin coated cover slips in a 12-wells plate with 60,000 cells/well and incubated for 48 h with 10 nM recombinant myostatin and control was without myostatin. Cells were washed with PBS and fixated with 4% performaldehyde before permeabilization with 0.1% Triton (T8532, Sigma Aldrich). Primary anti-mouse-GDF8 antibody (ab71808, Abcam) was applied 1:50 and incubated overnight. Donkey anti-rabbit Alexa 647 (A31573) was applied 1:800 and after 60 minutes incubation Hoechst 34580 (Sigma-Aldrich, ref 63493) 1:1000 was applied. Cover slips were washed with PBS three times and pasted on glass slides mounted with Prolong^TM^ Gold (P36930, Invitrogen). Pictures were taken with Confocal Microscopy.

### 4.10. Histological and Immunohistochemical Assessment of Cuffed Femoral Arteries

Formaldehyde fixed cuffed femoral arteries were paraffin-embedded and 5µm thick cross sections of arteries were stained to visualize vessel morphology. For quantification of intimal thickening, elastic laminae were visualized with Weigert’s elastin staining. For immunohistochemical analysis, the following antibodies were used: Myostatin (ab71808; Abcam, Cambridge, UK), BrdU (ab221240; Abcam), Ki-67 (ab16667; Abcam), αSMA (1A4 clone, Dako), Mac-3 (550292; BD Pharmingen). Images of stained slides were obtained using Panoramic 250 Flash III (3DHISTECH). All quantifications were performed on six sequential representative sections per vessel segment using image analysis software (Qwin, Leica) for Weigert’s elastin staining. All other quantifications were done using Panoramic Viewer software (3DHISTECH).

### 4.11. Bone Marrow-Derived Macrophage Isolation and Stimulation

After sacrifice, femur and tibia bones from 0.0 µg myostatin/day group (control group) and from 0.4 µg myostatin/day group were collected and proximal and distal ends were removed. Bones were flushed with PBS with 25 G needle (ref 300600, BD Microlance) and bone marrow was collected in 70 µm cell strainer (BD Biosciences). After collection of bone marrow, cell strainers were flushed with PBS and isolate was centrifuged. After removal of the supernatant, washing steps were repeated two times. Washed pellet was resuspended in ACK lysing buffer (A10492, GIBCO) and kept on ice for 3 min. Lysis was stopped with culture medium (RPMI Medium 1640 (ref 52400-025, GIBCO) with 25% Fetal Bovine Serum (F7524, Sigma) and 1% penicillin/streptomycin). After two more washing steps with PBS, bone marrow monocytes were counted and seeded at 8,000,000 cells per dish (10cm diameter, Falcon, ref 351029) in 10mL culture medium with 2 µL GM-CSF (ref 14-8331-80, eBiosciences). Monocytes of both groups were cultured either with or without in vitro treatment of 10 nM myostatin. All conditions were performed in duplicate.

After 5 days, medium was refreshed and at day 8 after isolation, monocytes were maturated into macrophages and 10ng/mL LPS (K-235, Sigma) was added to trigger a cytokine release. After 48 h of stimulation, supernatant was collected, snap-frozen in liquid nitrogen and stored at −80 °C. TRIzol was added and cell lysates were stored in −20 °C before RNA isolation.

### 4.12. Enzyme-Linked Immuno Sorbent Assay (ELISA) of Supernatant Bone Marrow-Derived Macrophages

ELISA analysis was performed following standard manufacturer’s protocol for murine TNFα (ref 558534, BD OptEIA). Duplo samples of cultured macrophages were taken and each condition was tested in duplicate and using Cytation5 (BIOTEK) absorbance was read at 450 and at 570 nM. TNFα concentrations were calculated from standard curve.

### 4.13. Statistical Analyses 

Data are presented as mean ± SEM. Indicated differences have the following levels of significance: *p < 0.05, ** *p* < 0.01, *** *p* < 0.001, **** *p* < 0.0001. All tests were performed with a significance level of α < 0.05.

One sample t-test was performed to test differences of treated groups that are expressed relative to the control treatment, which is set to 100%. This test was used in myostatin addition and in vivo experiments and functional assays.

Between specific groups, the presence of differences was assessed with Student’s t-test. These tests were used in comparison of PCNA expression in different treatment groups.

A Kruskal–Wallis test was used to identify possible differences between treatment groups for MAC3 staining.

## 5. Conclusions

In conclusion, myostatin inhibits expression of 14q32 microRNAs, as well as cell proliferation in VSMCs. In vivo, myostatin treatment also reduced 14q32 microRNA expression and VSMC proliferation in the femoral artery. However, 14q32 microRNA expression in bone marrow-derived macrophages remained unaffected after myostatin treatment, nor did we observe changes in macrophage infiltration into the lesion. Moreover, myostatin treatment did not affect postinterventional restenosis. Our findings underline the fact that restenosis is driven by two major components, both VSMC proliferation and local inflammation. Therefore, therapeutic strategies to reduce postinterventional restenosis should aim to target both processes simultaneously.

## Supplementary Materials

Supplementary materials can be found at https://www.mdpi.com/1422-0067/21/10/3508/s1.
