# Supplementary material for: Myostatin Inhibits Vascular Smooth Muscle Cell Proliferation and Local 14q32 microRNA Expression, But Not Systemic Inflammation or Restenosis
Source: Int J Mol Sci. 2020 May 15;21(10):3508. doi: 10.3390/ijms21103508 (PMC7278907; doi:10.3390/ijms21103508)
Supplement: Supplementary file 1 [file ijms-21-03508-s001.pdf]

# Myostatin Inhibits Vascular Smooth Muscle Cell Proliferation and Local 14q32 microRNA Expression, But Not Systemic Inflammation or Restenosis

**Eveline A.C. Goossens, Margreet R. de Vries, J. Wouter Jukema, Paul H.A. Quax and A. Yaël Nossent**

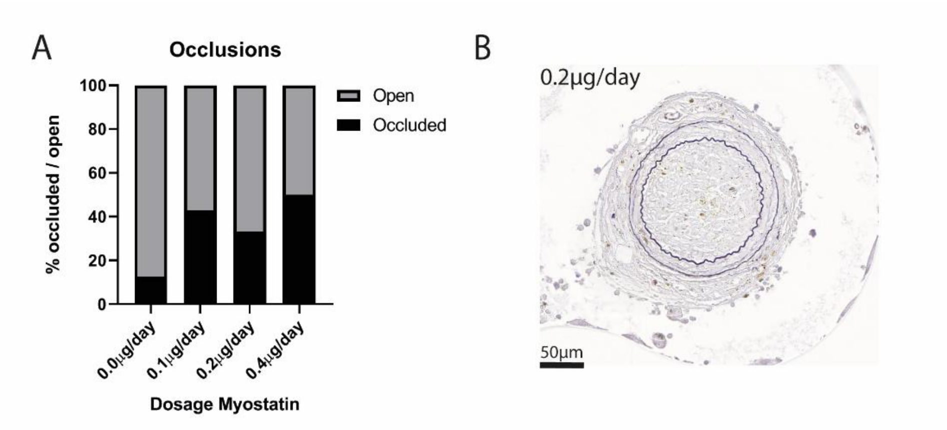


**Figure 1. A-B: Occluded cuffed femoral arteries in different treatment groups**. **A** – Occlusion percentages in different treatment groups. Control group N=8, 0.1µg/day N=7, 0.2µg/day N=6, 0.4µg/day N=8. **B** – Representative example of occluded cuffed femoral artery. Chi Square test did not show significant differences between groups.

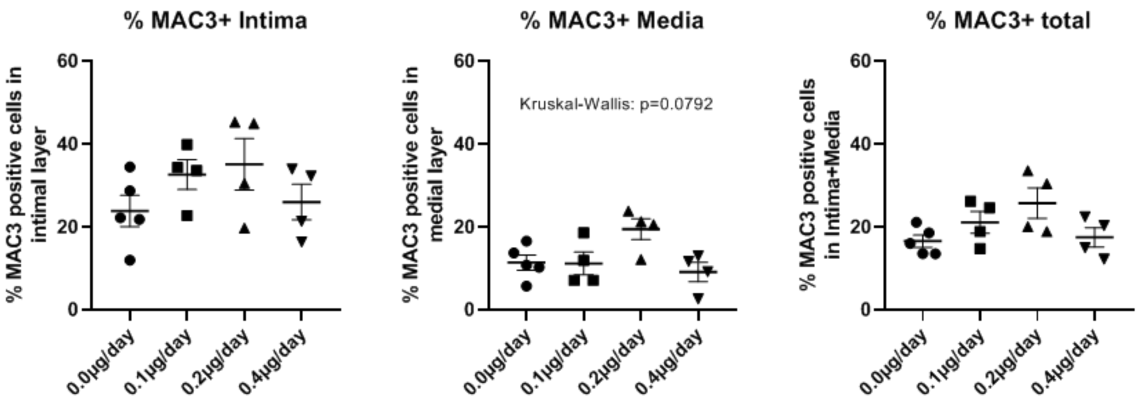


**Figure 2.** A-C: Quantification of MAC3+ cells in different layers as percentage of total amount of cells in different treatment groups. **A** – Percentage of MAC3+ cells in the intimal layer did not differ between treatment groups. **B** – Percentage of MAC3+ cells in the medial layer did not differ between treatment groups. **C** - Percentage of MAC3+ cells in the intimal and medial layer together did not differ between treatment groups. Kruskal-Wallis test did not show any significant differences between groups.

**Table 1.** qPCR Primer Sequences.

| | Forward Sequence | Reverse Sequence |
|---|---|---|
| MMU-PCNA | TCACAAAAGCCACTCCACTGT | CTTTAAGTGTCCCATGTCAGCA |
| MMU-MSTN | TGATCTTGCTGTAACCTTCCCA | TCATCGCAGTCAAGCCCAAAG |
| GAPDH | CACCACCATGGAGAAGGC | AGCAGTTGGTGGTGCAGGA |
